# Comment on “Acquired Tracheoesophageal Fistula after Esophageal Atresia Repair”

**DOI:** 10.4274/balkanmedj.galenos.2020.2020.6.85

**Published:** 2020-10-23

**Authors:** Vedat Akçaer

**Affiliations:** 1 Department of Pediatric Surgery, Trakya University School of Medicine, Edirne, Turkey

## To the Editor,

We read the article by Türer et al. (1) titled “Acquired Tracheoesophageal Fistula (TEF) After Esophageal Repair” published in the Balkan Med. J. and would like to share our thoughts on the article as well as alternative approaches to the subject.

Türer et al. (1) stated that the series of fistulas in their study were; two in the right bronchus, one in the cervical trachea, and one in the colon. They went on to define these fistulas as acquired TEF. However, when describing such complex cases, it may be more appropriate to use the term esophagotracheal fistula or esopfagopulmonic fistula instead of acquired TEF (2).

Authors suggested that there was no consensus on the timing and type of surgical treatment for recurrent TEF. However, the rate of recurrent TEF in end-to-end anastomosis was 5-15%, while in end-to-side surgeries, the rate increased from 25-40%. Therefore, end-to-end anastomosis should be the preferred procedure (3).

Authors looked at the case of three patients who required surgical treatment for acquired TEF. One patient’s fistula closed spontaneously while waiting for surgery. Muscle and pleural flaps were used in the surgical treatments. These flaps prevented the development of recurrent TEF, but it should be note that the use of these flaps can cause esophageal strictures by externally compressing the esophagus (2). Acquired TEF management is more difficult than that of congenital TEF (4). The treatment of acquired TEF can be in the form of endoscopic (minimally invasive) or open surgery. Endoscopic treatment, which is a safer method for both recurrent and acquired TEF patients, should be the first choice in recurrent TEF patients with esophageal atresia (3,4). This method is also cheaper, repeatable, and less aggressive (3). Moreover, endoscopic treatment is performed with deepithelization of the fistula and single or combined use of tissue adhesives (5). Tiscusol (fibrin glue) is a biological material that contributes to the closure of the fistula by increasing epithelization, revascularization, and reducing leukocyte infiltration (3).

They stated that anastomosis leakage, which consists mainly of saliva secretion and stenosis, is the most important risk factors in the etiology of recurrent TEF. Glycopyrrolate (Robinul), an anticholinergic drug with a saliva-reducing effect 5 to 6 times greater than atropine, ensures early closure of leak, reduces the need for mechanical ventilation, and helps to protect the patient's natural esophagus (6).
